# Introducing medical students to cultural psychiatry: perspectives and reflections on developing and delivering an elective module

**DOI:** 10.1192/bjb.2024.100

**Published:** 2025-10

**Authors:** Tahir Jokinen, Nawal Benachar, Arian Rahim

**Affiliations:** 1 Department of Global Health and Social Medicine, King's College London, UK; 2 South London and Maudsley NHS Foundation Trust, London, UK; 3 South West London and St George's Mental Health NHS Trust, London, UK; 4 Department of Education, Wolfson College, University of Oxford, UK; 5 Maidstone and Tunbridge Wells NHS Trust, Tunbridge Wells, UK

**Keywords:** Transcultural psychiatry, education and training, survey statistics (or survey methods), stigma and discrimination, qualitative research

## Abstract

Cultural sensitivity, competence and curiosity are essential for clinicians. To promote these, we developed an elective module in cultural psychiatry for medical students, consisting of eight seminars. In seminar eight, we used film clips to teach mental state examination. We comment on the development and delivery of the module, and offer a selection of student feedback. Cultural psychiatry could be better integrated into core medical school curricula, and we call for research to explore this.

Cultural psychiatry is beginning to receive more interest, but remains neglected in medical education. Perhaps much more so than any other medical specialty, psychiatry has an intimate relationship with the cultural field that it inhabits; a relationship that has been richly explored in the arts and humanities. Efforts have been made to encourage psychiatrists to think more explicitly about the role of cultural and transcultural factors in their work, as can be seen in the DSM cultural formulation.^1^ Nonetheless, medical education in the UK, as in elsewhere, has traditionally given very little space to the exploration of such issues.^2,3^ Undergraduate curricula may contain very little psychiatry at all, yet even postgraduate curricula for psychiatrists in training make only cursory mention of cultural psychiatry.

In recent years, there has been a growth in interest in the role of racial and ethnic biases, alongside other forms of discrimination, with increasing attention being given to making psychiatric services more inclusive and accessible to marginalised groups. Although this work is much needed and very welcome, there can be a tendency to limit understandings of cultural psychiatry solely to issues of discrimination, racism and inequality. Attention is drawn, for example, to the disproportionately high rates of involuntary detention of patients from minority ethnic groups;^4^ yet possible explanations for this are varied and speculative, and it has not been established that higher rates of detention are indeed related to racism or discrimination.^5^ Such topics can align closely with cultural psychiatry, and indeed fall within its remit, but we argue that the field is broader than this. In discussing ‘culture’, we align with the definition proposed by Clifford Geertz: ‘a system of inherited conceptions expressed in symbolic forms by means of which men communicate, perpetuate, and develop their knowledge about and attitudes toward life’.^6^ It is not, therefore, something that can be reified or reduced to a list of simple criteria or definitive meanings; rather, culture is alive and continually in a process of flux as people move constantly between cultural milieus, making sense of the world and others around them as they construct interpretations.

Cultural psychiatry, for us, includes all the myriad ways in which cultural factors affect people's experiences of mental illness; their understandings, conceptualisations and meanings; psychopathology and symptomatic presentations; and engagement with various aspects of treatment. Caregivers and professionals likewise operate in a cultural milieu of their own, and it is the interaction of all three parties and their distinct, even if sometimes overlapping, cultural milieus that makes cultural psychiatry so rich and fascinating, if daunting. Engagement with cultural psychiatry is therefore unavoidable for everyone working in mental health. We sought to inject some of this curiosity into medical students by developing an elective module, ‘Cultural Psychiatry’, on which we offer a commentary here.

The primary aim of this project was to develop medical students’ understanding of the complexity inherent to explorations of cultural factors in psychiatric practice, and equip them with clinically relevant skills. By doing so, we sought to integrate cultural competence into their broader clinical training. A secondary aim was to explore a novel way of teaching the mental state examination through film. Although the use of film to teach psychiatry has been theorised in general terms, we were able to find only one existing study that has employed film to specifically teach the mental state examination.^7^

## Method

We developed an elective module (student-selected component; one among a catalogue of options for students to select from) for second-year undergraduate medical students at a London medical school. We ran the module three times from 2020 to 2023. The course consisted of eight 3-h seminars over 16 weeks, facilitated by T.J. and N.B., for which students were expected to complete preparatory reading. Seminars began with a presentation by the facilitators to introduce the topic and key debates, and then moved to a mixture of unstructured discussion and group tasks. In the eighth and final seminar, we used short film clips to give students practical experience of conducting a mental state examination while reflecting on cinematic portrayals of mental illness in a range of diverse cultural contexts. Each student was expected to deliver an assessed individual oral presentation, and complete a written piece in the region of 2000 words of broad relevance to the module content. An outline of seminar content is presented in Table 1, and the films used in session eight, and corresponding psychiatric conditions, in Table 2. We collected quantitative and qualitative feedback on students’ experiences, perspectives and expectations through surveys at the beginning, mid-point and end of the course. Because of restrictions during the COVID-19 pandemic, the module was delivered online via videoconferencing in the first year, moving to majority in-person teaching for the subsequent years.

**Table 1:** Outline of module content across the eight seminars

Seminar title	Description of content
1. Basic principles of psychiatry	Outline diagnostic approaches and broad categories of mental illness, provide an overview of the mental state examination. History of psychiatry and key developments across the world. Exemplar seminar questions: How do we distinguish between ‘normal’ and ‘pathological’? How has the history of psychiatry contributed to practice today?
2. Introduction to cultural psychiatry	‘Culture’ as a category of analysis, insights from the humanities and social sciences. Brief overview of the various facets of cultural psychiatry. Exemplar seminar questions: What is the relevance of studying cultural psychiatry, and how, if at all, does it relate to practice? How do we define and understand the term ‘culture’, and what are the implications of the theoretical frameworks that we choose to apply? What are some of the academic approaches to studying culture, and what are their benefits and drawbacks?
3. Culture-bound syndromes	Psychiatric syndromes that have been considered unique to particular cultural contexts, comparing and contrasting these with more universally recognised mental health conditions. Explore whether they continue to be experienced in diaspora communities. Exemplar seminar questions: To what extent are human emotions culturally specific? To what extent are these syndromes genuinely limited to a specific cultural context? How far can they be considered unique conditions in their own right, as opposed to variations of universal mental disorders?
4. Culture and common mental illness	Differing ways in which more or less universally recognised mental disorders present in different cultural contexts, question the concept of a universal medical framework. Interrogate how culture affects presentation, patient experience, illness course and prognosis. Notions of illness and healing across different cultures. Exemplar seminar questions: To what extent are psychiatric diagnoses transferable and comparable across different cultural contexts? What are the common underlying themes that persist despite cultural variation?
5. Migration and global mental health	Impact of migration and displacement, diaspora communities and the acculturation process. Issues of stigma and access to healthcare. ‘Structural violence’, health policy and politics. Exemplar seminar questions: How does being from a migrant background affect patients’ presentation and experience of mental illness? What is the role of intergenerational trauma as a risk factor for mental illness? What is meant by ‘acculturation’ and what are some of the tensions inherent within this notion?
6. Religion and belief systems	Role of organised religion and shared belief systems in influencing patients’ experience of mental illness. Extremism and fundamentalism and the mental health aspects pertinent to these. Traditional healing practices including shamanism. Exemplar seminar questions: What is the relationship between spiritual, biological and psychological explanations of psychiatric conditions? What is the relationship between traditional healing practices and modern psychiatry?
7. Psychiatry, politics and society	Intersection of psychiatry with politics, society, and criminality. Notions of social and societal control. ‘Medicalisation’ in a range of historical and societal contexts. Exemplar seminar questions: What are the factors inherent in making a distinction between ‘normal’ and ‘pathological’? What is the role of politics in influencing the practice of psychiatry? How does politics inform structure, delivery and quality of healthcare?
8. Media, literature, film and the arts	Representations of mental illness in popular culture through a range of media and art forms and how these have varied across time and cultural context. Ways in which media, literature, film and the arts can influence attitudes toward mental illness and drive change in psychiatric practice. Exemplar seminar questions: To what extent is it possible to draw parallels between representations of mental illness in popular culture across different cultural contexts? In what ways can literature, film and the arts influence popular attitudes towards mental illness? In what ways can popular culture influence the practice of psychiatry?

**Table 2:** List of films used for teaching of mental state examination in seminar session eight, and corresponding psychiatric conditions

Film	Condition
A Beautiful Mind (2001)	Schizophrenia
Silver Linings Playbook (2012)	Bipolar affective disorder
Prozac Nation (2001)	Depression
My Name is Khan (2001)	Autism spectrum disorder
Girl, Interrupted (1999)	Personality disorders

## Results

Enrolment in the module ranged between 14 and 22 students, compared with a mean allocation of 8.4 to 9.7 students per elective module across the 3 years that the programme was run. All students that attended successfully completed the course, including the oral and written assessments. We present here some select perspectives and reflections derived from student feedback and our experiences of developing and delivering the module. Students came with minimal knowledge of psychiatry generally, and no prior engagement with cultural psychiatry. They found the volume of reading challenging in the beginning, and some were daunted by the prospect of engaging in complex discussions in a large seminar-group format. However, students gained confidence and competence as the course progressed, and many reflected at the end that they had found the readings and discussions enjoyable and stimulating once more accustomed to them. Illustrative quotations from student surveys are displayed in Table 3, organised under key themes: engagement and interest, challenges, essential learning and new perspectives. Seminars generated insightful, thoughtful discussion, and students were able to be curious and open in exploring perspectives and questions, and willing to offer ideas while remaining respectful of cultural milieus that they had little prior exposure to. Seminar eight (media, literature, film and the arts) was particularly well received. Students found the central task, mental state examination through film clips, to be a useful way of learning and practising a key psychiatric skill while thinking about the complexities and controversies inherent in cinematic portrayals of mental illness. Table 4 presents a summary of student feedback specific to seminar eight, including quantitative and qualitative elements.

**Table 3:** Selected illustrative quotations from student feedback on the module in surveys

Theme	Quotations
Engagement and interest	‘I found it very interesting to learn about how different cultures can affect the presentation and experience of mental illness in different people.’ ‘Made me more excited for our psych placements.’ ‘[Film] cannot be a substitute to teaching the MSE [mental state examination] on actual patients, but it is a fun and novel method of familiarising students with the examination and some of the issues involving psychiatric presentations in film.’ ‘I found it interesting hearing other people's response and opinions to certain topics.’
Challenges	‘The assignment was difficult given our knowledge levels and also time constraints, it felt like to do any topic justice would require a dissertation level of commitment.’ ‘I found case discussion in small groups the least useful because I felt we didn't get enough time to read and discuss the case fully.’
Essential learning	‘Highlighted the problems that can occur when two or more distinct cultures clash … look at when this has happened in history … learned how cultures can interact and interfere with one another and how the consequences of this continue to influence the way healthcare is delivered today.’ ‘Understanding problems of representation can help realise where we restrict access to services.’ ‘Made me think more about how movie makers sensationalise psychiatric symptoms for dramatic effect and the negative connotations this can have for real people with mental health conditions and the psychiatric service provider.’ ‘People can be hesitant to seek help or accept treatment from psychiatrists for many reasons, including religion, culture etc. It is important to understand the individual's understanding of their mental illness and try to come up with a treatment that both you and the patient are happy to try.’ ‘Overall how western secular teachings are not always applicable.’
New perspectives	‘There are no easy answers to many questions in this field and it is important not to jump to conclusions.’ ‘Learning more about your patient in general including their beliefs, upbringing and general background can only help improve the doctor-patient relationship.’ ‘Allowed us to reflect on our own practice … hopefully will make us better clinicians in the future.’ ‘Cultural psychiatry is broader than I thought it would be. Psychiatry is not universal.’

**Table 4:** Quantitative and qualitative feedback on seminar eight (media, literature, film and the arts)

Domain	Feedback
Effectiveness	94.8% found film an effective teaching medium.
Confidence	73.7% felt confidence in mental state examination was increased significantly.
Enjoyment	100% enjoyed the activity.
Usefulness	89.5% would find further sessions useful.
Future career planning	84.5% would be more likely to consider pursuing psychiatry.
Qualitative feedback	‘Really fun and engaging … a good introduction to mental state examinations.’ ‘Very enjoyable. It's often helpful to learn in different contexts, and films contribute to the idea of 3-dimensional patients.’ ‘Encouraged us to be analytical and think about mental health issues in a wide social context, a really useful learning task.’ ‘Was good to change up the format of the discussions in an interactive way.’ ‘I gained a deeper insight into the patient's perspective during a mental state examination.’

## Discussion

We believe that our experiences of developing and delivering this elective module demonstrate that cultural psychiatry can, and should, be more fully integrated into medical curricula. Over the 3 years that we offered the module, it remained a popular choice and students produced assessed work of a high standard. Some have gone on since to develop these into more comprehensive projects, including a culturally informed cognitive behavioural therapy mobile application for depression among Muslim patients. Medical school curricula can sometimes be superficial, with teaching tailored to single best answer questions. We aimed through this course to encourage students to engage on a deeper level with complicated topics that go beyond the limits of biomedicine. We were keen to emphasise that although there may not be any ‘right’ answers, that does not mean that we should not be thinking about challenging issues and asking difficult questions. Above all, we hope that the module contributed in a small way toward developing thoughtful and reflective future clinicians, who are curious and confident to engage with patients and caregivers from all manner of cultural backgrounds, and able to think sensitively about cultural and transcultural issues. These issues are not limited to psychiatry, and arise in every branch of medicine. We also suggest that the use of film can be an innovative and engaging way of teaching skills in mental state examination, but caution that this should be done sensitively, with attention to controversies and potential stigma.^7^ Psychiatric interview and examination skills are often a point of anxiety for medical students and trainees who may have little exposure during medical school; using the mode of film can provide a convenient, flexible alternative. Guided by a facilitator, this method allows students to practice in a safe space, with scope for the introduction of new terms and key psychiatric concepts to early learners. It also provides opportunity to explore the potentially negative aspects of cinematic portrayal of mental illness, bringing awareness to stigma and social issues, and helping create compassionate, culturally sensitive clinicians.

A key limitation of our module was that it was intensive and required a significant teaching commitment. Undergraduate curricula are already overly full, and it would be unrealistic to expect all students to engage with cultural psychiatry at the level of depth that we advocate. More thought needs to be given to how some of this key learning could be translated into a shorter form that could be incorporated into core curricula, without losing the nuance, sensitivity and curiosity that is central to the practice of cultural psychiatry, and indeed culturally sensitive medicine more broadly. By restricting our course to medical students, we also limited the range of professionals that we could reach. We suggest that mental health professionals of all backgrounds, including psychologists, nurses and social workers, would benefit from thinking deeply about cultural psychiatry; more thought needs to be given to incorporating this into the relevant curricula. Our commentary on the module lacks data to support our perspectives because it is intended more as a reflective piece. Research is needed to explore more systematically how cultural issues can be more effectively taught in psychiatric and broader curricula. Nonetheless, we hope that our thoughts can spark interest and further discussion.

## About the authors

**Tahir Jokinen** is an academic clinical fellow in psychiatry at the Department of Global Health and Social Medicine, King's College London, UK; and a core trainee in psychiatry with Lewisham Personality Disorder Service, South London and Maudsley NHS Foundation Trust, London, UK. **Nawal Benachar** is a core trainee in psychiatry with the Home Treatment Team, South West London and St George's Mental Health NHS Trust, London, UK; and an MSc student at the Department of Education, Wolfson College, University of Oxford, UK. **Arian Rahim** is a core trainee in surgery with the Ear, Nose and Throat Department, Maidstone and Tunbridge Wells NHS Trust, Tunbridge Wells, UK.

## Data Availability

The data that support the findings of this study are available from the corresponding author, T.J., upon reasonable request.
